# Book Review: The Animal Mind

**DOI:** 10.3389/fpsyg.2017.02030

**Published:** 2017-11-21

**Authors:** Alain Morin, Famira Racy

**Affiliations:** ^1^Department of Psychology, Mount Royal University, Calgary, AB, Canada; ^2^Department of Psychology, Adler University, Chicago, IL, United States

**Keywords:** mind, animals, self, self-awareness, thinking, feeling

TIME published a special edition on *The Animal Mind* (Kluger, 2017) that caught our attention, not as a result of the cute doggy face on its cover, but because of the subtitle “How they think, how they feel, how to understand them.” Unfortunately, given several problems with the edition, some of which are discussed below, none of these intriguing questions are truly answered. The book (more accurately: magazine or booklet, with only 96 pages) contains eight sections on multiple aspects of animals' overt and covert behavior—obviously including the animal mind (cognition, self-awareness, creativity), as well as group behavior (life in animal society), animals' skills at manipulating their environment (e.g., building nests), and themselves (e.g., camouflage) to survive, relationships (friendship, bonding), mourning (grief, sorrow), non-verbal communication and language use, mental illness (depression, obsessive-compulsive behavior), animal' rights, and our interactions with animals.

We certainly appreciate the effort put into this special edition, and we think that Kluger and his team succeeded in putting together an accessible and interesting collection of chapters, some scientifically well-balanced. To illustrate, the section on animal mourning contains numerous hints that some seemingly straightforward evidence of grief (e.g., female baboons carry dead babies for extended periods of time) may be inconsistent with other behaviors (e.g., they are mating at the same time). However, there are several key problems with *The Animal Mind* at the outset, hence we concentrate on the introduction (p. 4) and the first section (“Animals have brains, but do they have minds?,” pp. 6–17).

First, key terms are not defined. Critically, the edition pertains to the animal “mind” (used eight times in the aforementioned two sections), yet no definition is offered. Depending on what dictionary you consult or person you ask, “mind” may refer to numerous things, including the ability to be aware of the world and one's experiences, to think (e.g., to reason), and to feel (online Oxford Dictionaries) (art, n.1., 2017). If one decides to embrace this definition, then “mind” actually signifies both “consciousness” (awareness of one's environment) and “self-awareness” (knowledge of one's inner states) (Morin, 2006). Or “mind” may be defined as one's intellect, but then, what does “intellect” mean? Thus, is *The Animal Mind* special edition about consciousness (used 10 times, undefined), self-awareness (undefined), intelligence (together with “intellect” and “smarts,” used fourteen times, undefined), or something else? Other important yet undefined terms used in the special edition are awareness, subjective experience, cognition, thought, planning, reflecting, and creativity. There is a call in the scientific community to help stop misinformation and to increase reader awareness through defining key terms in context with discussions (Racy, 2015; Morin, 2017). Not knowing what these words mean makes it very difficult for the scientist or general readership to appreciate what the special edition is trying to say.

Second, in the Time edition (Kluger, 2017) as well as scientific literature (e.g., Gallup, 1985; Keenan et al., 2003), self-recognition in front of a mirror (Mirror Self-Recognition—MSR) is often equated with “awareness of self” (p. 16), “awareness of one's existence” (p. 11), or “distinction between self and others” (p. 14). This reasoning is problematic: the special edition uses MSR as evidence that some animals may possess self-awareness, yet this line of evidence has been seriously disputed by several researchers (Mitchell, 1993; Povinelli, 1995; Brandl, 2016; Saidel, 2016), including one of the current authors (Morin, 2003, 2010). MSR most likely simply presupposes awareness of one's body and has little to do with awareness of one's mental states; MSR does not provide evidence for the presence of self-reflection or the process of asking, “Is that me in the mirror?” Consequently, statements such as “Animals, the research *proves*, are creatures capable of reflection” (p. 4, emphasis added) will induce misunderstanding.

Third, even if MSR were to underlie full-blown self-awareness (which it doesn't, see above references), it has been observed only in a few non-human animals—in only two elephants as a case in point (Plotnik et al., 2006). The special edition gives the impression that all “Elephants, apes, and dolphins… pass the mirror test” (p. 14), yet only a very small sample of them have actually passed it. (Incidentally, some magpies also pass the mirror test—see Soler et al., 2014). Tiny samples, combined with the fact that the MSR test does not represent self-awareness, means there is not nearly enough evidence to claim that non-human animals are self-aware; any claim of this sort is speculation at best at this time.

Fourth, the aforementioned samples are biased. All animals tested for MSR and other mental abilities such as language were raised in captivity. Wild animals do not get exposed to human interactions or sophisticated life experiences that may artificially enhance their “mind”. Thus, many observations and conclusions presented in *The Animal Mind* edition mostly apply to captive animals.

We have seen popular sources of information such as those in Time Magazine consult what seems like the scientific literature, as evident in this special edition. We have also seen the influence of these popular disseminations, as well as fallacies in the scientific literature, in our classrooms, and in every day discussions. This is our critical reply to the state of scientific and popular knowledge production, dissemination, and consumption, using one small (yet highly influential) example from a re-emerging field of interest. We acknowledge that most writers of popular sources are not professional scientists and tend to take for granted the word of renowned researchers. This TIME special edition reflects a way of doing science that unfortunately is growing in our field.

## Author contributions

All authors listed have made a substantial, direct and intellectual contribution to the work, and approved it for publication.

### Conflict of interest statement

The authors declare that the research was conducted in the absence of any commercial or financial relationships that could be construed as a potential conflict of interest.
